# Valorization of canary seeds lipid fraction and defatted flour by supercritical carbon dioxide extraction

**DOI:** 10.3389/fnut.2026.1770930

**Published:** 2026-02-20

**Authors:** Esther Trigueros, Marina Villanueva, M. Teresa Sanz, Alba Esther Illera, Felicidad Ronda

**Affiliations:** 1 Department of Agriculture and Forestry Engineering, Food Technology, College of Agricultural and Forestry Engineering, University of Valladolid, Valladolid, Spain; 2 Research Institute on Bioeconomy - BioEcoUVa, ProCerealTech Group, University of Valladolid, Valladolid, Spain; 3 Department of Biotechnology and Food Science, Chemical Engineering Division, University of Burgos, Burgos, Spain

**Keywords:** canary seed, functional food, gel rheological properties, gluten-free, PUFAs, supercritical CO_2_, tocopherols

## Abstract

**Introduction:**

Canary seeds (*Phalaris canariensis* L.) are rich in starch, proteins, and lipids, especially polyunsaturated fatty acids, however their potential remains underexplored.

**Methods:**

This study evaluated supercritical CO2 extraction (SCCO2) to obtain valuable lipids and defatted flour, evaluating the chemical characterization of the extracted oils and a thorough assessment of the properties of the resulting defatted flours. Refined (RF) and whole (WF) flours were defatted by SCCO2 (41 ± 1 °C, 40 ± 1 MPa, 360–540 min) and compared with hexane extraction (HX) (80 °C).

**Results and discussion:**

The fatty acid profile of the lipid fraction of RF and WF revealed no significant differences, both being rich in mono- (14 g/100 g-oil) and polyunsaturated (76 g/100 g-oil) fatty acids. Unlike HX, SCCO2 preserved the tocopherol content in the extracted oil, with *γ*-tocopherol being the most abundant form. WF exhibited better hydration and surfactant properties than RF after defatting. Defatted flours showed increased peak (19–38%) and breakdown (20–45%) viscosities respect to the original flours due to lipids limiting water absorption. SCCO2-defatted flours showed the highest amylose retrogradation, with setback viscosity up to 26% higher than non-defatted samples. All flours studied showed the ability of forming gels with a predominantly elastic character. Gels made with SCCO2-extracted flours showed an increased elastic modulus (+20%) with respect to HX-extracted ones indicating an increased elastic behavior. In conclusion, SCCO2-extracted oil contained valuable polyunsaturated fatty acids and preserved tocopherols, whereas defatted flours improved functional and gelling properties, making them promising food thickening agents.

## 1 Introduction

Canary seed (*Phalaris canariensis* L.) is an annual cereal grain of the *Poaceae* family. Canada is the leading producer, accounting for 159,306 tons, followed by Thailand (36,469 tons), and Australia (36,114 tons) (1). Traditionally, canary seeds have been primarily used as birdfeed, as their siliceous trichomes on the seed hull, linked to esophageal cancer, makes them unsuitable for human consumption. Canada has recently developed novel hairless varieties safe for consumption (2). In 2015, these glabrous varieties were classified as Generally Regarded as Safe (GRAS) by the Food and Drug Administration (FDA), allowing them as novel food. Regarding its composition, starch (50–60%), with lower amylose content than wheat, constitutes its major component (3). The protein (19–23%) and lipid (7–9%) contents are higher than in other cereals such as barley, oat, and wheat, although fiber (2–6%) is lower (4). Its gluten-free proteins make it an excellent alternative for developing non-gluten products. Canary seeds also contain minerals, vitamins, phenolic compounds, and carotenoids, which make them a good source of essential micronutrients (3). Despite a global focus on palm, soybean, rapeseeds, and sunflower oils, canary seed oil offers high levels of essential fatty acids and a favorable polyunsaturated/saturated fatty acid ratio, associated with health benefits such as cholesterol reduction and the prevention of heart disease and atherosclerosis (5).

With the recent development of hairless varieties, research on their chemical composition, valorization, and potential applications remains limited. For instance, among the few published articles, Achouri et al. (6) investigated protein extraction from dehulled canary seed using various buffers and alkaline treatments, while Abdel-Aal et al. (7) employed wet milling with ethanol, water, and alkaline solutions to extract starch, protein, and lipid fractions. Moreover, a previous study by Náthia-Neves et al. (8) examined the impact of lipid removal on canary seed flour properties, conducting a lab-scale defatting process for 40-h using hexane.

Supercritical CO_2_ extraction is an environmentally friendly alternative to traditional methods employing large volumes of organic solvents. It consists of using CO_2_ under a temperature and a pressure exceeding its critical point, which provides unique properties, such as higher diffusivity, lower viscosity, and reduced surface tension, thereby enhancing mass transfer. In addition, the process leaves no solvent residue, allowing the collection of the raffinate without needing solvent removal steps (9).

Given the general research gaps in canary seeds and the lack of studies exploring supercritical CO_2_ to valorize their lipid fraction producing defatted flour, the aims of this study are to (1) assess the feasibility of supercritical CO_2_ extraction for oil recovery from canary seed flours under reproducible industrial conditions, (2) analyze the chemical composition of the oil fractions, (3) evaluate the chemical, functional, thermal, and rheological properties of the flour before and after defatting, and (4) compare these results with those obtained using conventional hexane extraction. Beyond these objectives, the novelty of this study lies in the application of supercritical CO_2_ extraction as a sustainable approach to simultaneously valorize canary seed oil and the resulting defatted flour. To the best of our knowledge, this is the first study to directly compare supercritical CO₂ and conventional hexane extraction, highlighting their impact on oil composition and flour functionality, and further supporting the potential industrial application of canary seeds as a novel food ingredient.

## 2 Materials and methods

### 2.1 Materials and reagents

#### 2.1.1 Materials

The hairless canary seed grains, corresponding to the CDC-Maria variety, were kindly supplied by Fitopal Company (Palencia, Spain). Samples were stored under refrigeration (4 ± 2 °C) until processing.

#### 2.1.2 Reagents

Isooctane, hexane and isopropanol (HPLC grade), NaOH, methanol, NaCl, BF_3_, methyl tricosanoate, and tocopherol isomer standards (*α*, *β*, *γ*, and *δ*) were obtained from Sigma-Aldrich. Corn oil was supplied by Koipe Asua (Córdoba, Spain).

### 2.2 Dehulling and grinding process

**Figure 1:**
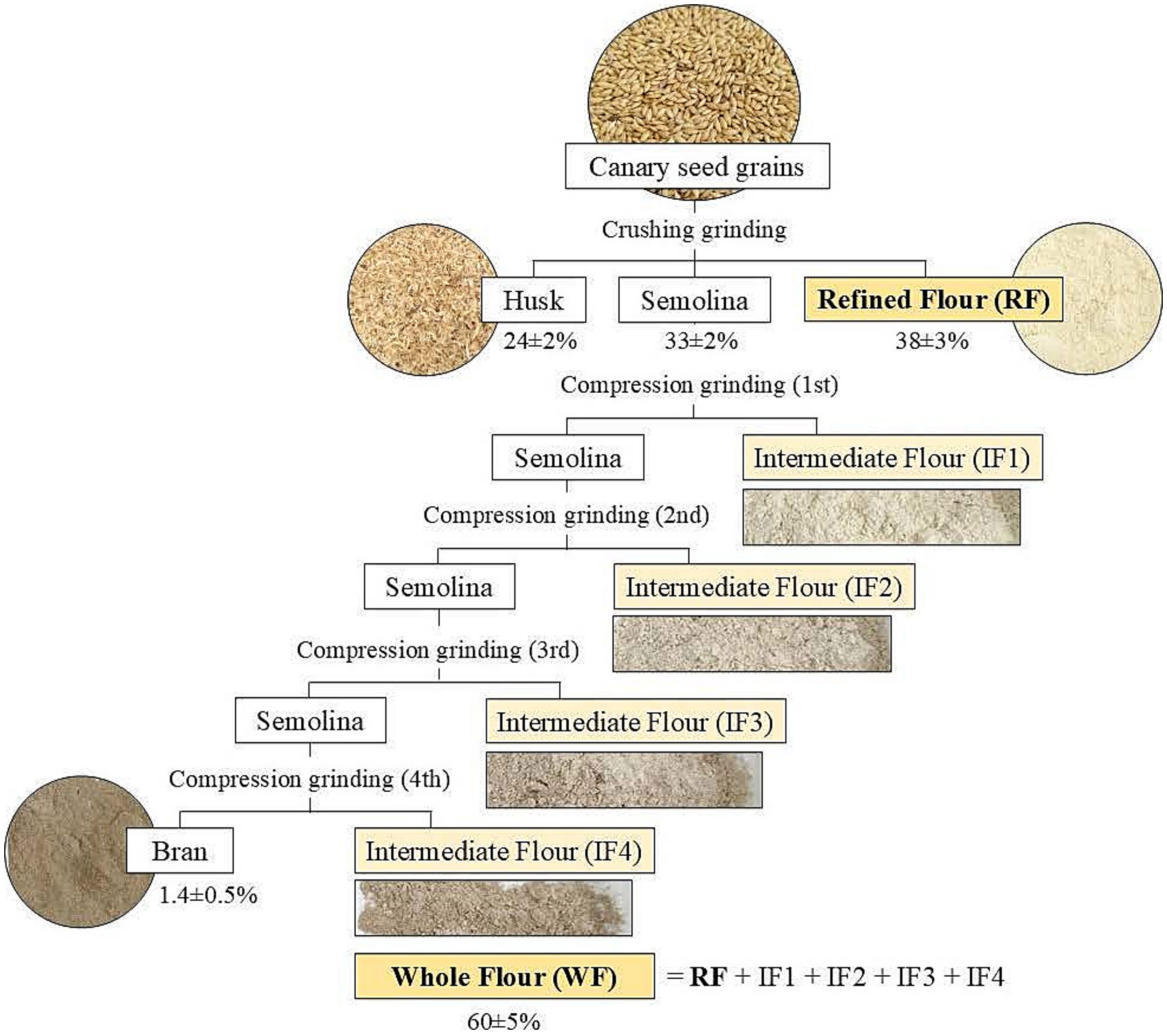
Scheme of the canary seed grains hulling and milling process to obtain the flours of interest: refined flour (RF) and whole flour (WF). The numbers represent the values of the extraction yields for the different fractions of interest.

To produce the flours examined in this study, the canary seed grains were dehulled and processed through multiple cycles of crushing and compression grinding using a CHOPIN CD1 mill (Chopin, Villenueve-la-Garenne Cedex, France). A scheme of the process, including yields, is shown in Figure 1. First, the canary seed grains underwent crushing grinding, which separated the husk, semolina, and refined flour (RF), the first flour evaluated in this study. To obtain the second flour of study, the semolina underwent four compression grinding cycles, producing four intermediate flours (IF1-4) which, combined with the initial RF, produce the whole flour (WF). A small stream of brand, representing less than 1.4% remained as a by-product.

### 2.3 Lipid extraction procedures

#### 2.3.1 Supercritical CO_2_ extraction (SCCO2)

**Figure 2:**
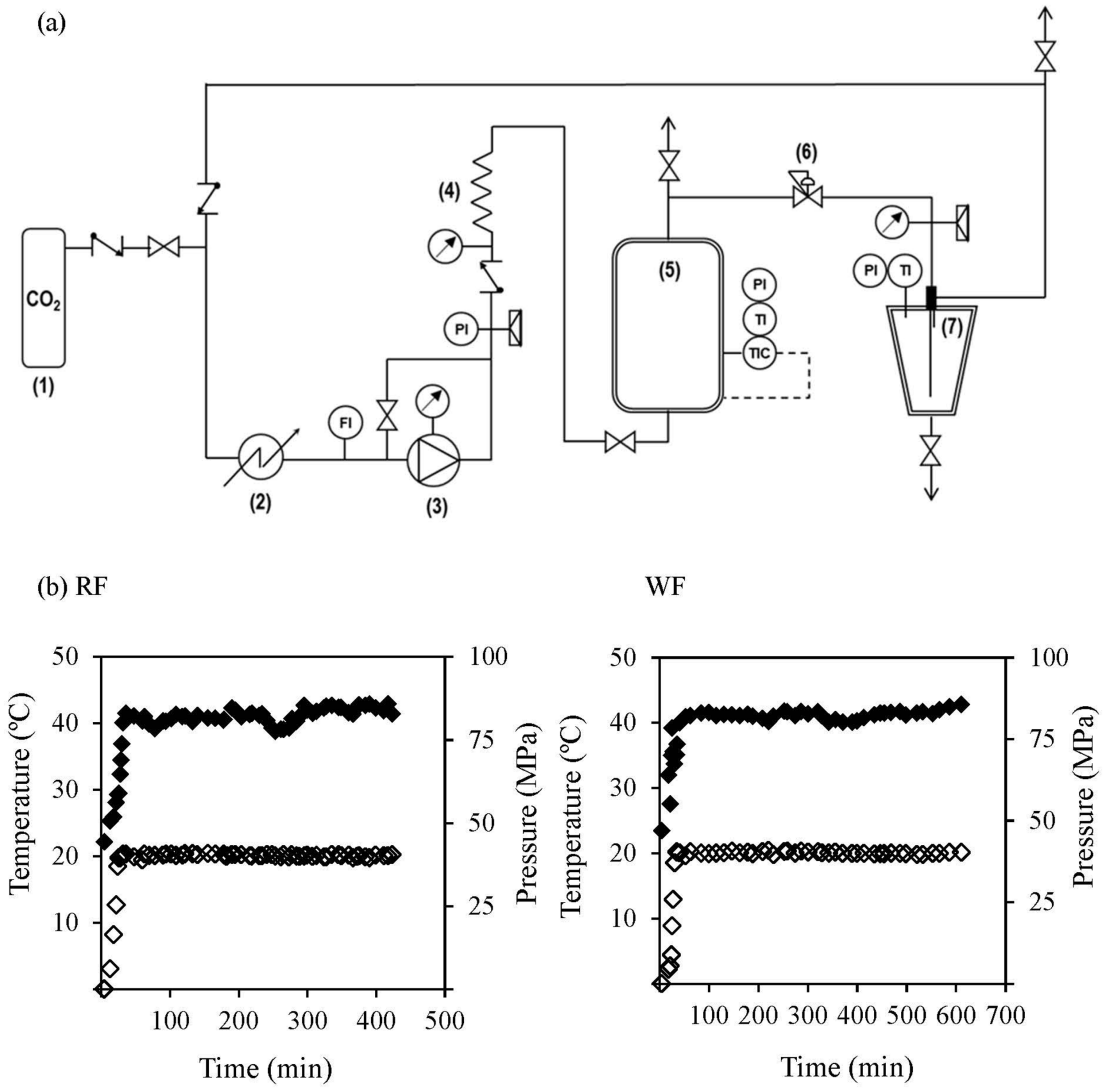
**(a)** Schematic diagram of the pilot-scale SCCO_2_ system employed for extracting the lipid fraction from the canary seed flours. Main elements: (1) Liquid CO_2_ reservoir; (2) cryostat; (3) pump; (4) heat exchanger; (5) extractor; (6) back pressure valve; (7) separator. FI, mass flow indicator; PI, pressure indicator; TI, temperature indicator; TIC, temperature indicator and controller. **(b)** Temperature (◆) and pressure (◇) profiles during extraction time for the refined flour (RF) and the whole flour (WF).

Lipid extraction using supercritical CO_2_ was performed in a pilot-scale system, as illustrated in Figure 2a. The setup included a diaphragm pump (EH1, LEWA), a stainless-steel extractor (2 L, 70 MPa max pressure, 200 °C max temperature), and a separator (1.1 L, 30 MPa max. Pressure, 120 °C max. temperature). Throughout the extraction process, control parameters were monitored using a Measurement and Data Acquisition System (DAS-8000, Desin Instruments). Temperature and pressure profiles inside the extractor are shown in Figure 2b.

During the process, temperature and pressure in the extractor were maintained at 41 ± 1 °C and 40 ± 1 MPa, respectively. Once these conditions were reached, a 30 min equilibrium period was initiated. Afterwards, the SCCO2 extraction started maintaining a CO_2_ flow rate of 12–15 kg/h. Throughout the extraction, the lipid fraction was collected in a separator maintained at 20 °C and 5 MPa. At the end of the extraction, the oil was stored at 4 °C until analysis protected from light to avoid oxidation. The extraction was considered complete when the weight of the lipid fraction stabilized. Due to the higher lipid content of WF compared to RF, the extraction durations were 540 and 360 min, respectively.

#### 2.3.2 Conventional extraction

For comparison with SCCO2, RF and WF samples were defatted by Soxhlet extraction using n-hexane at 80 °C for 4 h, after which no additional oil was collected in the vessel. This temperature ensured continuous solvent recirculation. The lipid fractions obtained from this process were stored at 4 °C and protected from light until analysis.

### 2.4 Oils characterization

*Fatty acid profile.* The fatty acid profile of the oil extracted by SCCO2 from both RF and WF was analyzed following the Official Method AOAC 991.39 (10). Briefly, the fatty acids were derivatized to methyl esters and analyzed using Gas Chromatography with a Flame Ionization Detector (GC-FID, Agilent 6,890 N), equipped with an Omegawax-320 column (30 m × 0.32 mm), with helium as carrier gas. Injection and detector temperatures were 250 °C. The oven temperature increased from 180 °C to 200 °C (1 °C/min – 1 min hold), followed by a ramp to 220 °C (5 °C/min – 15 min hold). The results were calculated as the percentage of relative area obtained from duplicates.

*Tocopherol profile.* The tocopherol profile was determined using High-Performance Liquid Chromatography with Diode-Array Detection (HPLC-DAD, HP1100) coupled with an ACE 5 Silica column (250 × 4.6 mm). The mobile phase consisted of hexano:isopropanol (99,1, v/v), in isocratic mode. The flow rate was maintained at 1 mL/min, and the column temperature was set at 25 °C. An injection volume of 50 μL was used, and the detection was performed at 296 nm for 15 min. Identification was achieved by comparing retention times and spectra with standards under identical experimental conditions, and quantification was carried out through calibration curves prepared with a mixture of the four tocopherol-isomer standards (*α*, *β*, *γ*, and *δ* isomers). All analyses were conducted in triplicate.

### 2.5 Characterization of canary seed flours

#### 2.5.1 Chemical composition

Moisture content of the flours was measured following the Official AACC Method 44-19.01, lipids were quantified following the Official AACC Method 30-25.01, and ash content was determined with the Official AACC Method 08-01.01 (11). Proteins were determined by combustion following the UNE-EN ISO 16634-1 (12) applying a nitrogen-to-protein conversion factor of 5.7 (13). Total carbohydrates were determined by difference to 100%. Total fiber was measured using an enzymatic-gravimetric method in accordance with the Official Method AOAC 985.29 (14). All analyses were conducted in triplicate.

#### 2.5.2 Particle size distribution (PSD)

The PSD of the flours was assessed using a laser diffraction particle size analyzer (Mastersizer 2000, Malvern Instruments Ltd., Malvern, UK) paired with a Sirocco dry powder feeder. Results were reported as the diameters at which 10% (D_10_), 50% (D_50_ or median diameter), and 90% (D_90_) of the particles have smaller size, and as the PSD dispersion ((D_90_-D_10_)/D_50_). Experiments were performed in triplicate.

#### 2.5.3 Techno-functional properties

Water absorption capacity (WAC), oil absorption capacity (OAC), water absorption index (WAI), water solubility index (WSI), and swelling power (SP) were assessed on a dispersion of 5 g flour/100 mL, and foam capacity (FC) and foam stability (FS) were measured at a 2 g/100 mL, following the methods outlined by Abebe et al. (15). Emulsifying activity (EA) and emulsion stability (ES) were evaluated according to Vicente et al. (16). The results were expressed as follows: WAC and OAC in *g*_water_/*g*_dried-flour_ and *g*_oil_/*g*_dried-flou_, respectively, WAI in *g*_sediment_/*g*_dried-flour_, WSI in *g*_soluble-solids_/100 *g*_dried-flour_, SP in *g*_sediment_/*g*_insoluble-solids_, FC as the foam volume in mL, FS as the percentage of foam after 60 min with respect to FC, EA as the percentage in volume of emulsion formed relative to the initial volume, and ES as the percentage in volume of emulsion after heating relative to the initial volume. All measurements were performed in triplicate.

#### 2.5.4 Pasting properties

The pasting properties of both non-defatted and defatted flours were assessed using a Rapid Visco Analyser (RVA) model 4500 (Perten Instruments, Australia). The Standard 1 temperature profile from the Official Method AOAC 76-21.01 (17) was applied. From the pasting curves, generated in triplicate, the pasting temperature (PT), peak viscosity (PV), peak time (Pt), trough viscosity (TV), breakdown viscosity (BV), final viscosity (FV), and setback viscosity (SV) were obtained.

#### 2.5.5 Rheological properties

The rheological properties of gels prepared at a concentration of 3.5 g (based on 14% moisture content) in 25 g of distilled water following the procedure described in the Section 2.5.4 were determined using dynamic oscillatory tests. Tests were performed using a Kinexus Pro + rheometer (Malvern Instruments Ltd., Malvern, UK), equipped with parallel plate geometry (40 mm diameter) and a 1 mm working gap. Gels were placed between the plates, allowed to relax for 5 min at 25 °C. Strain sweeps were carried out from 0.1 to 1,000% strain (1 Hz) to determine the linear viscoelastic region (LVR), while frequency sweeps were performed from 10 to 1 Hz (1% strain). The frequency sweep data were fitted to the power-law model (18). All samples were measured by triplicate.

#### 2.5.6 Differential scanning calorimetry (DSC)

Flour thermal properties, including gelatinization and retrogradation transitions, were analyzed using a differential scanning calorimeter (DSC3, STARe-System, Mettler-Toledo, Switzerland) following the method described by Vicente et al. (16). A ratio of 30 g solid to 70 g water was used for the analysis. The first scan, on fresh samples, assessed the gelatinization and dissociation of the amylose-lipid complex. A second scan, conducted after samples being stored for 7 days at 4 ± 2 °C, evaluated the melting of recrystallized amylopectin and the dissociation of the reversible peak of the amylose-lipid complex. Both scans were performed from 0 to 120 °C, at a heating rate of 5 °C/min. For each scan, the enthalpy (∆*H*) was measured in J/g of dry matter, along with the peak temperature (*T*_p_) in °C, and the gelatinization temperature range (∆*T*_gel_), calculated as the difference between *T*_end-set_ and *T*_on-set_. The degree of retrogradation was calculated as the area of retrogradation related to that of gelatinization and expressed as percentage. Experiments were conducted in duplicate.

### 2.6 Statistical analysis

All results are presented as mean ± standard deviation (SD), obtained from at least two replicates. Statistical analysis was performed using Statgraphics Centurion 19 (Bitstream, Cambridge, MN, USA). To determine significant differences, analysis of variance (ANOVA) with least significant difference (LSD) test (*p* < 0.05) was conducted.

## 3 Results and discussion

### 3.1 Oil characterization

The fatty acid profiles of the oils obtained by SCCO2 are shown in Table 1. The values reported are only those obtained by SCCO2, as no differences have been reported in the literature in the fatty acid profiles of oils obtained by SCCO2 and conventional hexane extraction (19, 20).

**Table 1:** Fatty acid and tocopherol profiles of oil extracted from canary seed flours including those defatted by supercritical CO_2_ extraction (RF-CO2, WF-CO2) and by conventional hexane extraction (RF-HX, WF-HX).

Fatty acids (g/100 g oil)	RF-CO2	WF-CO2

Butyric acid (C4:0)	0.010 ± 0.002^a^	0.009 ± 0.003^a^
Caproic acid (C6:0)	0.007 ± 0.001^a^	0.010 ± 0.001^a^
Lauric acid (C12:0)	nd	0.0138 ± 0.0001
Myristic acid (C14:0)	0.036 ± 0.001^a^	0.0430 ± 0.0001^a^
Pentadecylic acid (C15:0)	0.0178 ± 0.0001^a^	0.022 ± 0.001^a^
Palmitic acid (C16:0)	5.77 ± 0.08^a^	6.07 ± 0.03^a^
Palmitoleic acid (C16:1)	0.094 ± 0.001^a^	0.100 ± 0.001^a^
Margaric acid (C17:0)	0.0538 ± 0.0002^a^	0.03 ± 0.04^a^
Stearic acid (C18:0)	2.72 ± 0.03^a^	2.77 ± 0.02^a^
Oleic acid (C18:1n9)	12.4 ± 0.2^a^	12.66 ± 0.05^a^
Vaccenic acid (C18:1n7)	0.81 ± 0.02^a^	0.84 ± 0.01^a^
Linoleic acid cis (C18:2n6)	54.2 ± 0.7^a^	54.6 ± 0.2^a^
*γ*-Linolenic acid (C18:3n6)	3.54 ± 0.12^a^	3.36 ± 0.01^a^
*α*-Linolenic acid (C18:3n3)	16.2 ± 0.2^a^	16.08 ± 0.06^a^
Stearidonic acid (C18:4n3)	1.09 ± 0.02^a^	1.05 ± 0.01^a^
Arachidic acid (C20:0)	0.95 ± 0.03^a^	0.97 ± 0.01^a^
Eicosadienoic acid (C20:2n6)	0.076 ± 0.001^a^	0.0755 ± 0.0003^a^
Dihomo-*γ*-linolenic acid (C20:3n6)	1.1 ± 0.9^a^	0.6 ± 0.5^a^
Behenic acid (C22:0)	0.35 ± 0.03^a^	0.38 ± 0.01^a^
Lignoceric acid (C24:0)	0.3 ± 0.2^a^	0.16 ± 0.02^a^
Nervonic acid (C24:1n9)	nd	0.03 ± 0.05
SFAs	10.2 ± 0.2^a^	10.5 ± 0.1^a^
MUFAs	13.3 ± 0.1^a^	13.6 ± 0.1^b^
PUFAs	76.3 ± 1.2^a^	75.7 ± 0.5^a^
*ω*-6	58.9 ± 1.2^a^	58.6 ± 0.5^a^
ω-3	17.3 ± 0.2^a^	17.1 ± 0.1^a^

Tocopherol content (mg/kg_oil_)	RF-HX	RF-CO2	WF-HX	WF-CO2	Analysis of variance		
					Flour (F1)	Defatting process (F2)	F1 × F2
*α*-tocopherol	2.7 ± 0.3^a^	16 ± 2^b^	4.5 ± 0.7^a^	29 ± 2^c^	***	***	***
*β*-tocopherols	25 ± 1^a^	36 ± 1^b^	45 ± 3^c^	53 ± 2^d^	***	***	ns
*γ*-tocopherols	60 ± 1^a^	124 ± 2^c^	68 ± 3^b^	133 ± 2^d^	***	***	ns
*δ*-tocopherol	nd	nd	nd	nd	–	–	–
Total tocopherol content	87 ± 1^a^	176 ± 4^c^	118 ± 1^b^	214 ± 2^d^	***	***	*

Data are presented as mean ± standard deviation (*n* = 3). SFAs, saturated fatty acids; MUFAs, monounsaturated fatty acids; PUFAs, polyunsaturated fatty acids; nd, non-detectable. Values with different letters in the same row are significantly different at *p* < 0.05. Analysis of variance: *p < 0.05, **p < 0.01, ***p < 0.001, ns, not significant.

The fatty acid profile of the oils obtained from both RF and WF were not significantly different. The most abundant fatty acids were linoleic acid, followed by *α*-linolenic and oleic acid, belonging to the omega-6, omega-3, and omega-9 series, respectively. The saturated fatty acids (SFAs) palmitic and stearic acid, along with the omega-6 *γ*-linolenic acid, were also found at high levels in canary seed flours. Other fatty acids were found in smaller proportions including the SFAs arachidic, dihomo-*γ*-linolenic, behenic, and lignoceric acids, the monounsaturated (MUFA) vaccenic acid, and the polyunsaturated (PUFA) stearidonic acid. Lauric acid and nervonic acid were not detected in RF oil. Overall, oils from both flours were characterized by a low SFAs content compared to a high proportion of unsaturated fatty acids (UFAs = MUFAs + PUFAs), with an UFAs:SFAs ratio of 9, exceeding the ratio found in olive oil of 5.8 (13). The high UFAs content in canary seed oil makes it beneficial for human nutrition and health. This fraction primarily consisted of PUFAs (~76%), making these oils a rich source of healthy fats. These findings agree with those reported by other authors. For instance, Abdel-Aal et al. (21) analyzed the fatty acid profile of oil from CDC Calvi and CDC Cibo canary seed varieties, identifying linoleic acid as the major fatty acid, followed by oleic, palmitic, *α*-linolenic, and stearic acid. Moreover, they determined a mean UFAs:SFAs ratio of 6.4. In another study, Tunisian *Phalaris canariensis* seeds were found to contain primarily linoleic acid, followed by oleic, palmitic, and linolenic acids, and smaller amounts of stearic, arachidic, palmitoleic, myristic and eicosanoic acids. This composition resulted in a high PUFAs:SFAs ratio, superior to that of soybean oil, largely due to the higher content of MUFAs, especially oleic acid. The high content of PUFAs, particularly linoleic and oleic acids, classifies canary seed oils among “oils low in palmitic acid and high in oleic and linoleic acids”, along with sunflower, safflower, olive, palm, and sesame oils (5). While excessive consumption of SFAs is associated with adverse health effects such as increased LDL-cholesterol and cardiovascular risk, MUFAs and especially PUFAs, are recognized as health-promoting nutrients, exhibiting positive effects on atherosclerosis, cardiovascular health, diabetes, autoimmune disorders, inflammatory processes, and other diseases (5, 13). In this context, the content of *α*-linolenic acid in canary seed oil, both from refined and whole flour (16.1–16.2%, Table 1), was higher than that reported for several commonly consumed vegetable oils, including canola (9%), soybean (6.6–8.03%), wheat germ (4.68%), corn and moringa (1.08%), olive (0.67–0.78%), avocado (0.73%), sesame (0.51%), grape seed (0.33%), sunflower (0.28%), and walnut (0.05%) (22–24). Comparable values were observed for hempseed oil (15.77%) (22), whereas substantially higher levels have been reported for linseed oil (53.6–56.42%), a widely recognized source of *α*-linolenic acid (22–24). Therefore, canary seed oil can be classified as a moderate-to-high source of *α*-linolenic acid relative to commonly consumed vegetable oils, contributing to its nutritional value as a precursor of the long-chain omega-3 fatty acids EPA and DHA. This fatty acid profile makes *P. canariensis* oil an exceptional source of healthy fats, with potential applications in nutrition, cosmetics, and pharmaceuticals.

The tocopherol profiles of oils from RF and WF obtained by SCCO2 and conventional extraction have been presented in Table 1. Although fatty acid profile was not affected by the defatting process, the tocopherol content and profile were influenced. Vitamin E, found as tocopherols and tocotrienols in *α*, *β*, *γ*, and *δ* forms, is a fat-soluble vitamin distributed in many vegetal foodstuffs with antioxidant and anticancer properties (25). In this study, total tocopherol content ranged from 87 to 214 mg/kg_oil_. Among tocopherols, *γ*-tocopherol was the most abundant, followed by *β*- and *α*-tocopherol, while *δ*-tocopherol was undetectable. In general, higher levels of tocopherols were observed in the oils extracted from WF compared to RF, showing the type of flour (F1) a significant effect (*p* < 0.001). This result was expected, as the milling process enriched the oil content in WF, leading to higher lipid and tocopherol levels. Regarding the defatting process, oils obtained by hexane extraction exhibited significantly lower (*p* < 0.05) contents of *α*-, *β*-, and *γ*-tocopherol in both RF and WF samples, compared to those obtained by SCCO2, observing a significant effect of the defatting process (F2) (*p* < 0.01). The *α*-tocopherol content in oils obtained by SCCO2 was 5.9 (for RF) and 6.4 (for WF) times higher than in oils from hexane extraction. This enhancement is attributed to the lower temperatures used in SCCO2 extraction compared to conventional hexane extraction, highlighting SCCO2 as a promising method for obtaining high-value lipid fractions from canary seed flours while preserving tocopherol content. Similar findings were reported by Wang et al. (20), who noted lower tocopherol yields (0.03 mg/g_lipid-extract_) from sorghum grains using recirculated solvent extraction with hexane at 68 °C, observing higher extraction yields by decreasing the temperature (up to 0.52–0.62 mg/g). Additionally, in line with this study, SCCO2-oils had higher tocopherol content (0.36–0.70 mg/*g*_lipid-extract_).

Oils extracted by SCCO2 contained significant *α*-tocopherol levels (16–29 mg/kg_oil_), exceeding those of other vegetal sources. For example, the *α*-tocopherol content in the WF-CO2 sample exceeded that found in by-products of red currant, gooseberry, grape, pomegranate, watermelon and canary melon (26). Moreover, all the oils obtained in the present study from canary seed flours had higher *β*-tocopherol content (25–53 mg/kg_oil_) than in these other vegetal sources, but generally lower *γ*-tocopherol levels (60–133 mg/kg_oil_). Limited studies have assessed the vitamin E content in canary seed grains. Malunga et al. (27) determined vitamin E content across four canary seed varieties, exhibiting levels of *α*-tocopherol below 0.666 mg/100 g, identifying it as a poor source of tocopherols. According to EU Regulation 1169/2011, a food is considered with a significant amount of vitamin E when contains 15% or more of the daily reference intake (DRI) [European (28)]. Vitamin E content in WF-CO2 oil would supply 24.2% of the DRI for *α*-tocopherol and over 100% for total tocopherols, making it valuable for the development of functional foods with significant vitamin E content.

### 3.2 Characterization of canary seed flours

#### 3.2.1 Chemical composition

The chemical composition of canary seed flours is presented in Table 2. Canary seed flours were primarily composed of carbohydrates, with values of 74.4 ± 1.7 g/100 g dry basis (db) for RF and 70.3 ± 2.4 g/100 g db for WF, being comparable to other cereals such as wheat and oats (29). Lower values were previously reported by other authors for different canary seed varieties (13, 29). RF contained 5.8% more carbohydrates than WF (*p* < 0.05), since RF consists mainly of starch-protein matrix from the endosperm, while WF includes also germ and bran, which contain other components (30). Canary seeds have been recognized as one of the most protein-rich cereals, comparable to legumes (31), and containing significant essential amino acids (13). In this study, the protein content was 17.2 ± 1.5 g/100 g db for RF and 19.0 ± 1.7 g/100 g db for WF. The higher protein level in WF could be due to the presence of the germ and bran, richer in protein, compared with RF (30). These results are consistent with literature indicating higher protein content in whole grains compared to refined flours (13, 30). Oil in canary seeds is primarily found in the germ, endosperm, and aleurone layer (30). Therefore, the lipid content in RF (6.1 ± 0.6 g/100 g db) was significantly lower (*p <* 0.05) than in WF (8.1 ± 1.6 g/100 g db). The oil content observed is consistent with previous reports (4, 6) and is higher than in pulses like black gram and chickpea, and cereals like rice, wheat, and millet (32). Regarding canary seed flours derived from defatting, no significant differences were observed between the two defatting methods. Both HX and SCCO2 reduced the lipid content to less than 1.7 g/100 g db in both refined and whole flours, with a mean oil extraction yield of 81 ± 2% relative to the initial oil content in the flours. Moisture content was affected by the defatting process, with non-defatted flours having a moisture content of 14.6 ± 0.9 g/100 g for RF and 13.6 ± 0.8 g/100 for WF. Defatting with hexane resulted in a significant reduction in moisture content, of −58% in RF and −73% in WF. In contrast, SCCO₂ caused only a small decrease of about 6–7% in both samples. Urbizo**-**Reyes et al. (2) reported a 27% reduction in moisture content after defatting by mechanical pressing. Other studies on soy (33) and quinoa (34) flours found moisture reductions of 16–18% with hexane, attributed to the solvent’s high evaporation temperature (69 °C) which contribute to drying. Fiber was the least abundant component in both RF and WF, which aligns with the results of Abdel et al. (3, 35), although, as could be expected, WF contained a significantly (*p* < 0.05) higher content (2.4 ± 0.5 g/100 g db) than RF (< 1.2 ± 0.2 g/100 g db).

**Table 2:** Chemical composition of canary seed flours including refined flour (RF) and whole flour (WF), and their corresponding samples defatted by conventional hexane extraction (RF-HX, WF-HX) and supercritical CO_2_ extraction (RF-CO2, WF-CO2).

Composition	RF	RF-HX	RF-CO2	WF	WF-HX	WF-CO2
Moisture content	14.6 ± 0.9^e^	6.2 ± 0.3^b^	13.6 ± 0.2^d^	13.6 ± 0.8^d^	3.7 ± 0.0^a^	12.8 ± 0.1^c^
Protein	17.2 ± 1.5^a^	17.4 ± 1.6^a^	18.2 ± 1.6^a^	19.0 ± 1.7^a^	19.0 ± 1.7^a^	19.4 ± 1.7^a^
Lipid	6.1 ± 0.6^b^	1.1 ± 0.7^a^	1.0 ± 0.3^a^	8.1 ± 1.6^c^	1.6 ± 0.1^a^	1.7 ± 0.1^a^
Ash	2.3 ± 0.4^a^	2.6 ± 0.3^ab^	2.7 ± 0.6^ab^	2.6 ± 0.2^ab^	2.9 ± 0.4^ab^	3.3 ± 0.4^b^
Carbohydrates	74.4 ± 1.7^b^	78.9 ± 1.7^d^	78.1 ± 1.8^cd^	70.3 ± 2.4^a^	76.5 ± 1.8^bcd^	75.6 ± 1.8^bc^
Fiber	< 1.2 ± 0.2^a^	< 1.2 ± 0.2^a^	< 1.2 ± 0.2^a^	2.4 ± 0.5^b^	< 1.2 ± 0.2^a^	< 1.2 ± 0.2^a^

Data are presented as mean ± standard deviation (*n* = 3). Values with different letters in the same row are significantly different at *p* < 0.05. Moisture content was expressed in g/100 g of sample and the other composition parameters are expressed in g/100 g on a dry basis (db).

#### 3.2.2 Particle size distribution

**Figure 3:**
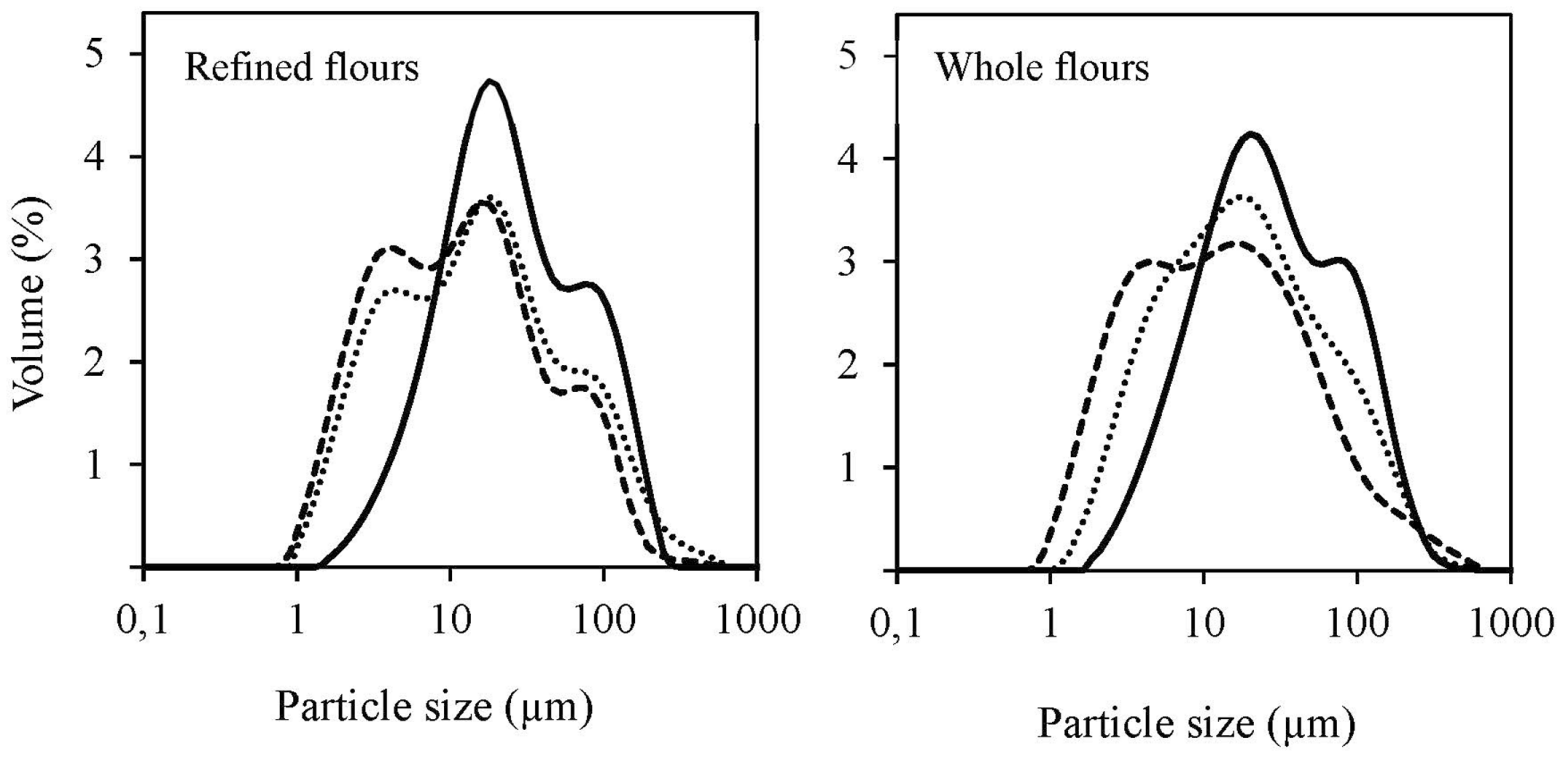
Particle size distribution of canary seed flour samples, both non-defatted (RF: refined flour; WF: whole flour) (—) and their corresponding defatted through conventional hexane extraction (RF-HX, WF-HX) (---) and supercritical CO2 extraction (RF-CO2, WF-CO2) (···).

The particle size of flours directly affects the techno-functional properties of food products. Particle size parameters and distribution curves for both non-defatted and defatted flours are shown in Table 3 and Figure 3, respectively. Non-defatted flours exhibited a bimodal PSD, with RF having a greater volume percentage of finer particles than WF. While both flours showed similar distributions in the coarse fraction, WF had significantly larger D_50_ and D_90_ values, due to the presence of small amounts of germ and bran. Bran in canary seeds appears as thin flakes with diameters ranging from 0.3 to 1.0 mm (3), which explains the larger particle sizes observed in WF compared to the bran-free RF sample. Between the defatted samples, the PSDs of RF-HX and RF-CO2 were similar (Figure 3), retaining the two peaks observed in RF but with reduced volume contributions. In RF defatted samples, a new peak appeared at smaller particle sizes (0.80 to 6.88 μm), also reflected in the particle size parameters (Table 3). The D_10_, D_50_, and D_90_ values significantly decreased in defatted RF flours, in greater extent in RF-HX than RF-CO2 samples, meanwhile the size dispersion increased by 37 and 42%, respectively. For WF defatted samples, peak differentiation was greater (Figure 3). Unlike the RF defatted samples, only two peaks were identified in the WF-HX sample, with the peak corresponding to the largest particle sizes missing (−38% in D_90_). In contrast, the WF-CO2 sample exhibited a three-peak distribution, although the peaks were softer and appeared as slight shoulders. These results are consistent with previous studies, such as Nahimana et al. (36), who found smaller particle sizes in defatted sweet yellow lupin proteins due to hot defatting processes disrupting protein aggregates. This could be an advantage when using defatted flours, as finer particle sizes improve dough rheological properties, while coarser flours result in higher baking losses, tougher crumbs and higher chewiness (3).

**Table 3:** Particle size distribution and techno-functional properties of canary seed grain flour samples, both non-defatted (RF, refined flour; WF, whole flour) and their corresponding defatted through conventional hexane extraction (RF-HX, WF-HX) and supercritical CO_2_ extraction (RF-CO2, WF-CO2).

Samples	RF	RF-HX	RF-CO2	WF	WF-HX	WF-CO2	Analysis of variance		
							Flour (F1)	Defatting process (F2)	F1 × F2
Particle size distribution									
D_10_ (μm)	6.2 ± 0.1^d^	2.2 ± 0.1^a^	2.5 ± 0.1^b^	6.2 ± 0.2^d^	2.2 ± 0.1^a^	3.5 ± 0.1^c^	*	***	*
D_50_ (μm)	22 ± 1^d^	11 ± 1^a^	14 ± 1^b^	24 ± 1^e^	11 ± 1^a^	16 ± 1^c^	***	***	ns
D_90_ (μm)	99 ± 4^c^	67 ± 5^a^	89 ± 5^b^	109 ± 6^d^	68 ± 10^a^	95 ± 5b^c^	ns	***	ns
(D_90_-D_10_)/D_50_	4.3 ± 0.2^a^	5.9 ± 0.6^b^	6.1 ± 0.4^b^	4.2 ± 0.3^a^	5.7 ± 1.0^b^	5.4 ± 0.3^b^	ns	ns	ns
Functional properties									
WAC (g/g)	0.56 ± 0.01^a^	0.68 ± 0.03^d^	0.65 ± 0.01^c^	0.60 ± 0.01^b^	0.73 ± 0.01^e^	0.66 ± 0.01 ^cd^	***	***	*
OAC (g/g)	0.98 ± 0.01^c^	0.83 ± 0.01^a^	1.04 ± 0.03^d^	1.01 ± 0.02^d^	0.88 ± 0.01^b^	1.02 ± 0.02^d^	*	***	*
WAI (g/g)	5.7 ± 0.9^a^	5.8 ± 0.3^ab^	6.6 ± 0.6ab^c^	6.3 ± 0.2^abc^	7.1 ± 0.6^c^	6.7 ± 0.1^bc^	*	ns	ns
WSI (g/100 g)	0.78 ± 0.05^b^	0.63 ± 0.02^a^	0.68 ± 0.04^ab^	1.31 ± 0.03^e^	0.98 ± 0.06^c^	1.16 ± 0.16^d^	***	***	ns
SP (g/g)	5.8 ± 0.9^a^	5.8 ± 0.3^a^	6.3 ± 0.1^ab^	6.4 ± 0.2^ab^	7.1 ± 0.6^b^	6.7 ± 0.1^b^	**	ns	ns
EA (%)	1.6 ± 0.4^a^	16.9 ± 1.6^c^	4.1 ± 0.4^b^	15.6 ± 1.3^c^	20.0 ± 1.5^d^	26.8 ± 0.4^e^	***	***	***
ES (%)	0	0	0	0	0	0	–	–	–
FC (mL)	1.5 ± 0.7^a^	6.1 ± 0.7^c^	3.9 ± 1.1^b^	3.0 ± 0.5^b^	9.2 ± 0.7^d^	5.2 ± 0.8^c^	***	***	ns
FS (%)	0^a^	32 ± 9^b^	30 ± 8^b^	0^a^	41 ± 9^b^	36 ± 1^b^	ns	***	ns

WAC, water absorption capacity; OAC, oil absorption capacity; WAI, water absorption index; WSI, water solubility index; SP, swelling power; EA, emulsifying activity; ES, emulsion stability; FC, foam capacity; FS, foam stability; D_10_, diameter at which 10% of the particles have a smaller size; D_50_, median diameter; D_90_, diameter at which 90% of the particles have a smaller size; (D_90_–D_10_)/D_50_: size dispersion. Values with different letters in the same line are significantly different at *p* < 0.05. Analysis of variance: *p < 0.05, **p < 0.01, ***p < 0.001, ns, not significant.

#### 3.2.3 Techno-functional properties

The hydration, foaming and emulsifying properties of canary seed flours are shown in Table 3. In general, the techno-functional properties exhibited higher values in the WF samples than in the RF samples. Regarding hydration properties, WF showed increases of 7–11% in WAC, WAI, and SP and of 64% in WSI compared to RF, suggesting a greater capacity for water interaction probably due to the higher levels of dietary fiber and other hydrophilic constituents. The ability of the flour to form emulsions and foams was strongly influenced by the type of flour and its composition. The EA and FC of WF were approximately 1 and two times higher than those of RF, respectively. However, none of the non-defatted flours were able to maintain emulsion or foam stability. Defatting process had a notably impact on the functional properties of the flours. The WAC increased - after defatting, with higher increases in HX samples (21–22%) than in those obtained by supercritical CO_2_ extraction (10–16%). This may be attributed to the smaller PSD in hexane-defatted flours, as smaller particles tend to absorb more water due to their increased surface area, which enhances water interaction (37). An increase in WAC is related to better food processing by avoiding liquid loss and favoring the preservation of texture, nutrients, and bioactive compounds (8). Consistent with these findings, numerous authors have documented enhanced absorption capacities of flours following SCCO2 extraction of soy (33) and wheat (38). The WAI and SP values did not change significantly because of defatting, regardless of the method used. In contrast, the WSI decreased by 8–24%, with a greater reduction observed in HX flours. These defatted samples also exhibited the greatest variation in OAC values with reductions of 15 and 13% for RF-HX and WF-HX respectively, meanwhile RF-CO2 and WF-CO2 showed a slight/not significant increase with respect to the initial values of RF and WF samples. For all flours, regardless of flour type or defatting process, the OAC value was higher than the WAC. This indicates the lipophilic behavior of canary seed flour, as previously reported by Rikal et al. (31). The most significant change after defatting was the marked improvement in EA, FC and FS of defatted flours. The EA significantly increased (*p* < 0.05) although the effect was dependent on the type of flour, the defatting process and the double interaction flour x defatting process (*p* < 0.001). Hexane extraction led to the highest increase in RF samples (10-times higher EA values), while for WF samples, SCCO2 showed the highest effect (+72%). None of these samples led to stable emulsions. Foaming capacity significantly increased (*p* < 0.05) by 2–4 times in all defatted samples, with HX flours showing the highest values regardless of flour type. Removing fat from canary seed flours increased the FS from zero to 30–41%, regardless of flour type and defatting process, related to the higher protein and lower lipid content.

#### 3.2.4 Pasting properties

**Figure 4:**
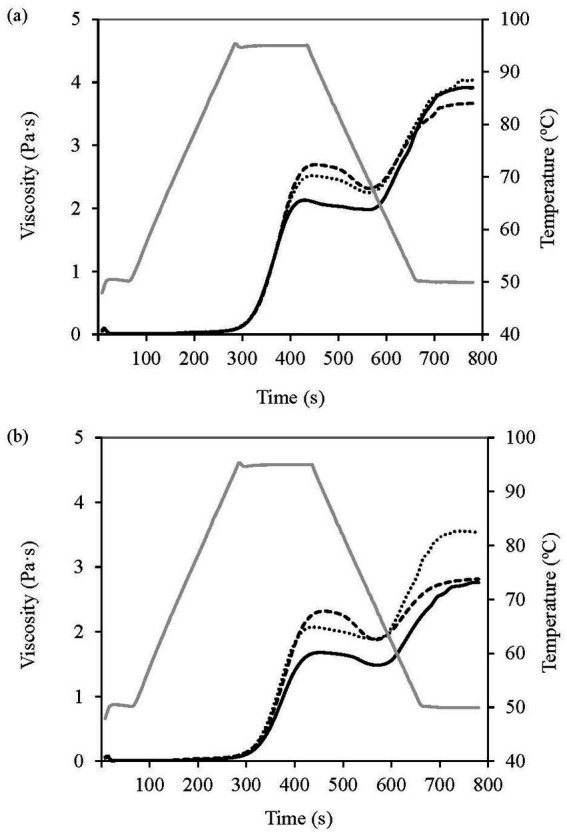
Pasting profiles of canary seed flours, **(a)** Refined flour and **(b)** Whole flour, including non-defatted (—) and defatted flours using conventional hexane extraction (---) and supercritical CO_2_ extraction (···). The temperature profile (—) is represented on the secondary axis.

The pasting parameters are presented in Table 4, and the pasting curves in Figure 4. The PT, or temperature at which the viscosity of starch slurries begins to increase during heating, and the Pt, representing the time to reach maximum viscosity, were significantly higher in WF than RF. For the PT, significant differences (*p* < 0.05) were observed depending on the flour type (F1), but not on the defatting process (F2). The PT and Pt values were higher than those reported for other cereals, such as waxy maize, wheat, tapioca, corn starch, and pea starch (3, 21, 39, 40). This may be explained by the high protein content of the canary seed flours examined in this study, which limits the swelling of starch granules and increases the temperature at which viscosity begins to rise.

**Table 4:** Pasting and rheological properties of canary seed grain flour samples, both non-defatted (RF, refined flour; WF, whole flour) and their corresponding defatted through conventional hexane extraction (RF-HX, WF-HX) and supercritical CO_2_ extraction (RF-CO2, WF-CO2).

Samples	RF	RF-HX	RF-CO2	WF	WF-HX	WF-CO2	Analysis of variance		
							Flour (F1)	Defatting process (F2)	F1 × F2
Pasting properties									
PT (°C)	94.67 ± 0.03^a^	94.70 ± 0.00^ab^	94.73 ± 0.06^abc^	94.80 ± 0.05^c^	94.76 ± 0.06^bc^	94.68 ± 0.03^a^	*	ns	*
PV (Pa·s)	2.13 ± 0.03^c^	2.69 ± 0.03^f^	2.53 ± 0.02^e^	1.68 ± 0.01^a^	2.32 ± 0.02^d^	2.07 ± 0.02^b^	***	***	**
Pt (min)	7.2 ± 0.1^a^	7.5 ± 0.1^cd^	7.4 ± 0.2b^c^	7.5 ± 0.1^cd^	7.7 ± 0.1^d^	7.3 ± 0.1^ab^	*	***	**
TV (Pa·s)	1.98 ± 0.03^c^	2.32 ± 0.02^e^	2.25 ± 0.04^d^	1.49 ± 0.02^a^	1.88 ± 0.05^b^	1.89 ± 0.02^b^	***	***	*
BV (Pa·s)	0.96 ± 0.09^b^	1.39 ± 0.03^e^	1.25 ± 0.07^d^	0.83 ± 0.04^a^	1.18 ± 0.04^c^	1.00 ± 0.03^b^	***	***	ns
FV (Pa·s)	3.91 ± 0.12^c^	3.67 ± 0.13^b^	4.00 ± 0.20^c^	2.76 ± 0.08^a^	2.81 ± 0.11^a^	3.53 ± 0.11^b^	***	***	**
SV (Pa·s)	2.75 ± 0.08^d^	2.51 ± 0.14^c^	2.87 ± 0.08^d^	1.99 ± 0.06^b^	1.87 ± 0.06^a^	2.50 ± 0.06^c^	***	***	**
Rheological Properties									
G_1_’ (Pa)	787 ± 30^c^	743 ± 5^b^	887 ± 36^d^	815 ± 1^c^	665 ± 16^a^	797 ± 18^c^	***	***	ns
a	0.015 ± 0.001^b^	0.025 ± 0.001^d^	0.010 ± 0.002^a^	0.018 ± 0.002^c^	0.024 ± 0.002^d^	0.023 ± 0.001^d^	***	***	***
G_1_” (Pa)	41.5 ± 1.0^c^	36.5 ± 0.5^b^	41.1 ± 0.4^c^	43.7 ± 2.2^d^	32.3 ± 1.2^a^	41.0 ± 0.5^c^	***	***	***
b	0.278 ± 0.002^b^	0.327 ± 0.006^d^	0.317 ± 0.002^c^	0.259 ± 0.009^a^	0.322 ± 0.005^cd^	0.287 ± 0.005^b^	***	***	**
(tan *δ*)_1_	0.053 ± 0.002^c^	0.049 ± 0.001^ab^	0.046 ± 0.002^a^	0.054 ± 0.003^c^	0.049 ± 0.002^ab^	0.051 ± 0.001^bc^	*	ns	*
c	0.263 ± 0.003^b^	0.302 ± 0.005^c^	0.307 ± 0.003^c^	0.241 ± 0.009^a^	0.298 ± 0.005^c^	0.264 ± 0.006^b^	***	***	***
*τ*_max_ (Pa)	179 ± 6^d^	129 ± 1^c^	206 ± 4^e^	91 ± 1^a^	111 ± 2^b^	132 ± 2^c^	***	***	***
Crossover (Pa)	228 ± 10^c^	216 ± 11^c^	263 ± 8d	115 ± 5a	171 ± 16^b^	175 ± 4^b^	***	**	**

PT, pasting temperature; PV, peak viscosity; Pt, peak time; TV, trough viscosity; BV, breakdown viscosity; FV, final viscosity; SV, setback viscosity; G_1_’, elastic modulus; G_1_”, viscous modulus; (tan *δ*)_1_: loss tangent; a, b, and c, exponents quantifying the dependence degree of the dynamic modulus and the loss tangent with the oscillation frequency (1 Hz) after fitting data to the power law model; *τ*_max_, maximum stress tolerated by the sample. Values with different letters in the same line are significantly different at *p* < 0.05. Analysis of variance: *p < 0.05, **p < 0.01, ***p < 0.001, ns, not significant.

PV, determined by the maximum swelling of starch granules, was always reached well after the start of the 95 °C hold period. The RF sample exhibited a PV value that was 21% above that of WF. This could be explained by the lower lipid content in RF (Table 2), as the lipid fraction limits water absorption and granule swelling (41). The PV values obtained for RF and WF were comparable to those previously reported for CDC Maria, C05041 (39), CDC Calvi, and CDC Cibo (21) canary seed varieties. After defatting, PV significantly increased (*p* < 0.05) for both RF and WF flours, demonstrating the role of lipids in hindering the swelling process. Nevertheless, when compared to other cereals, defatted flours from RF displayed similar PV values to corn starch and higher than wheat (21, 39, 40).

Both RF and WF, showed similar BV values to other reported canary seed varieties, but higher than wheat starch (39), and notably lower than those of waxy maize, tapioca, and pea starches (40). BV values, in general low, significantly (*p* < 0.05) increased after defatting, with the highest values observed in the HX samples, accounting for 45 and 42% of increase in RF-HX and WF-HX compared to RF and WF, respectively. These findings indicate that the great capacity of canary seed flours to withstand stress and heat is partially decreased after the defatting process.

SV indicates the starch gelling ability or retrogradation tendency (39). WF samples exhibited significantly lower values than RF (*p* < 0.05) due to its higher fat and fiber content. The highest values were found after SCCO2 defatting, surpassing those of the corresponding original flours, being especially notable the increase in WF-CO2 sample (26%) with respect to WF. On the contrary, HX reduced significantly SV values in both types of flours. These findings are consistent with previous studies, where the SV of wheat flour was higher in the SCCO2-defatted sample compared to hexane-defatted and non-defatted flour (38). The differences between the two defatting processes could be due to the higher temperatures used during hexane defatting process compared to SCCO2 (80 °C vs. 41 °C), which may promote a physical modification involving changes in starch structure (34). Similar results were reported by Irani et al. (39), who found comparable SV values for two canary seed varieties (CDC Maria and C05041) to those obtained in this study. Compared to other cereals, only pea starch showed similar SV values, while waxy maize, tapioca, corn, and wheat starch exhibited lower values, indicating lower gelling and retrogradation tendencies (21, 40). This rise in viscosity and gelling capacity in defatted flours may make them useful as thickening agents in food dispersions.

#### 3.2.5 Rheological properties of gels

**Figure 5:**
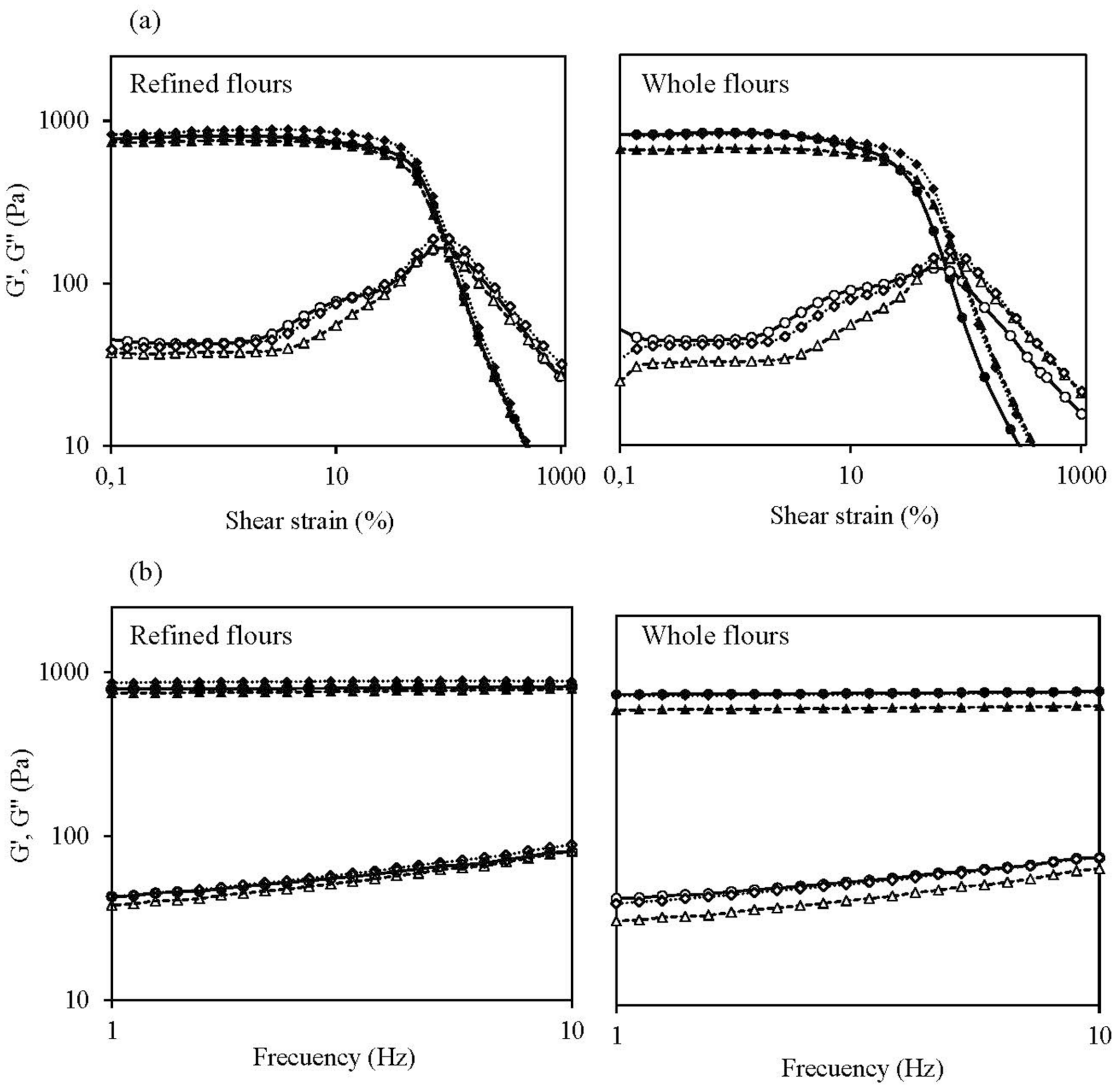
**(a)** Strain and **(b)** Frequency sweeps of the gels made with canary seed flours both refined and whole flours, including non-defatted (●○) and defatted flours using conventional hexane extraction (△▲) and supercritical CO_2_ extraction (◆◇). G’ is represented by full symbols and G” by empty symbols.

The parameters obtained from the rheological tests performed on gels with both non-defatted and defatted flours are presented in Table 4. This table presents the maximum stress value that the gel could withstand before rupture within the LVR, *τ*_max_, and the crosspoint (G′ = G′′) obtained from strain sweep tests, as well as the parameters G_1_’, G_1_,” (tan *δ*)_1_, and the exponents *a*, *b*, and *c* derived from fitting the power law model to the frequency sweep data within the LVR (0.898 < *R*^2^ < 0.997, data not shown). The corresponding frequency sweep and the strain sweep plots are shown in Figure 5.

To date, there is little evidence concerning the rheological properties of canary seed flour. In general, both the type of flour (F1) and the defatted process (F2), significantly influenced the gel’s viscoelasticity (*p* < 0.05), apart from F2 for the (tan *δ*)_1_ parameter, which showed no significant effect (Table 4). Additionally, significant interaction effects (F1 × F2) were observed for all rheological parameters except G_1_’, indicating that the impact of defatting process varied depending on the type of flour (Table 4). All gel samples exhibited predominantly elastic behavior within the LVR, with G_1_′ > G_1_′′ and (tan *δ*)_1_ values well below 1, implying a well cross-linked network structure. They also showed high stability, with very small dependence of G’ on frequency as can be concluded from the very low values of the exponent “a,” well below 0.1 (Table 4), and the small slope of the mechanical spectra (Figure 5b). No significant differences were observed in the elastic modulus (G_1_’) between RF and WF flours. However, G_1_’ significantly increased (*p* < 0.05) in RF-CO2 sample (13%), while no significant differences were observed between WF-CO2 and WF. By contrast, defatting with hexane resulted in reduced gel elasticity, with G_1_’ decreasing significantly by 6% for RF-HX and 18% for WF-HX compared to RF and WF, respectively. This reduction is associated with the decrease in FV and may be explained by the higher temperatures applied during the hexane defatting process compared with the SCCO2 process and the annealing process associated as described above. These results suggest that the rheological properties of the gels were influenced by both the presence and composition of the lipid fraction in flours, which could inhibit amylose leaching during gelatinization and its retrogradation by forming amylose-lipid complexes, thereby weakening the continuous phase and leading to a reduction in the viscoelastic moduli. The removal of most of the lipids can increase the friction among the starch granules, potentially enhancing G’ (42), as observed for RF-CO2 flour. The gel made with WF exhibited a significantly higher viscous modulus, G_1_,” than those made with RF (44 Pa vs. 42 Pa, respectively); however, this difference was not able to change significantly the value of (tan *δ*)_1_ that was mainly affected by the high values of G_1_’ in both RF and WF samples. Lipid removal led to a significant reduction in the viscous modulus (G_1_”) especially in the HX-defatted flours (−12% for RF-HX and −26% for WF-HX with respect to the original samples). In general, the gels made with defatted flours showed a significantly lower (tan *δ*)_1_ which indicates a better cross-linked network structure and a stronger solid-like character.

The low values of *a* exponent for flours, 0.015 for RF and 0.018 for WF, indicated minimal frequency dependence of the elastic modulus, G_1_’, suggesting the formation of stable gel structures. The *b* exponent, associated with the frequency dependence of viscous modulus (G_1_”), was significantly lower for WF (0.259) than for RF (0.278).

Regarding the maximum stress tolerated by samples within the LVR (*τ*_max_), the value was higher for RF- than WF-samples, increasing significantly after SCCO2-defatting, with increases of 60 and 19% in RF-SCCO2 and WF-SCCO2 samples with respect to HX-defatted counterparts. The higher *τ*_max_ values denote greater gel stability against shear, as higher stress levels are required to disrupt their structure and transition to a viscous state (8). The crossover point, which indicates the stress for the transition from predominantly elastic-like to viscous-like behavior, followed a parallel evolution to *τ*_max_ and was also significantly higher for RF than WF, probably due to the disruption and weakening effect that fibre can cause to the gel structure. The most stable gels were also obtained from samples defatted by SCCO2, which stresses at the crossing point increased by 15%, in RF-SCCO2, and 52%, in WF-SCCO2, with respect to their original RF and WF corresponding samples.

#### 3.2.6 Thermal properties

**Figure 6:**
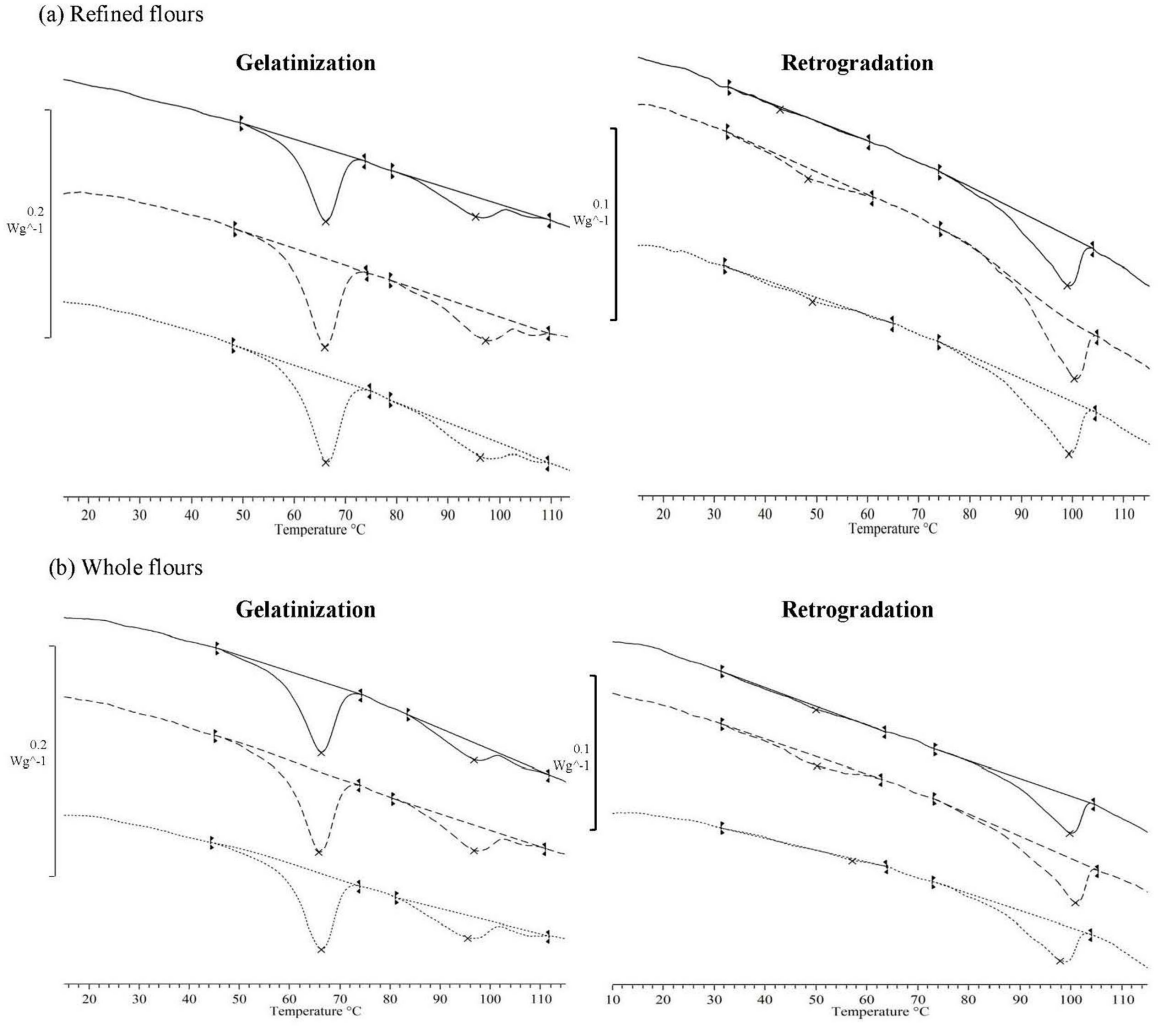
Differential scanning calorimetry (DSC) thermograms of canary seed flours, **(a)** Refined flours and **(b)** whole flours, including non-defatted (—) and defatted flours using conventional hexane extraction (---) and supercritical CO_2_ extraction (···).

The thermal properties of both non-defatted and defatted flours, as determined from the first gelatinization and the second retrogradation scans, are presented in Table 5. Thermograms are shown in Figure 6. During the gelatinization scan, two endothermic peaks were observed, as previously reported for canary seed flours (35, 39). The first peak, associated with starch gelatinization, appeared at a *T*_p_ of 66.0 ± 0.2 °C, with no significant effect of the type of flour or defatting process. However, the gelatinization temperature range (∆*T*_gel_) differed significantly between RF and WF. In WF, ∆*T*_gel_ was 1.0 °C wider because *T*_on-set_ decreased (−1%) while *T*_end-set_ increased (+1%), expanding the interval at both ends. This behavior can be attributed to the higher fiber content of WF, which absorbs and restricts the amount of water available for gelatinization and delays the melting of amylopectin crystallites in starch granules (43). The gelatinization temperatures found in the present study are higher than those of wheat flour, and are in line with previous reports on canary seed flours (35, 39). However, our gelatinization temperature range was half, indicating more uniform crystals formation in our canary seed samples. This could be attributed to differences in the canary seed cultivar and chemical composition, such as lipid and fiber content. The gelatinization enthalpy (∆H_gel_) significantly increased (*p* < 0.05) after defatting. In RF-CO2 and WF-CO2 samples ∆H_gel_ increased by 18 and 13% with respect to RF and WF, while for RF-HX and WF-HX this increase was smaller, 12 and 9%, respectively. This denoted a more stable starch structure in defatted samples which require more energy to destroy the crystalline area (44), highlighting the crucial role of lipid, amylose, and amylopectin interactions within the starch structure in determining the gelatinization properties of the flours. The differences between SCCO2- and HX-defatted flours could be attributed to the higher temperatures applied during conventional (hexane) defatting compared with the SCCO2 process. These higher temperatures may have promoted an “annealing” or slight “pre-gelatinization” effect, leading to a lower gelatinization peak area, as previously observed in defatted quinoa samples treated with hexane compared with those treated with SCCO2 (34). The small second peak, that appeared at 95–97 °C with a small ∆H, of ~ 3 J/g dry matter regardless of the type of flour and the presence/absence of fat, was probably due to the disruption of the amylose-lipid complex (43). It showed for all samples a small ∆H, around ~3 J/g dry matter, regardless of the type of flour and the presence/absence of fat. This suggests that the lipids involved in the amylose-lipid complex could not be removed during defatting processes, indicating that they were already bound to the starch within the granule.

**Table 5:** Thermal properties of canary seed grain flours, including both non-defatted (RF: refined flour; WF: whole flour) and the defatted flours through conventional hexane extraction (RF-HX, WF-HX) and SCCO2 (RF-CO2, WF-CO2).

Sample	RF	RF-HX	RF-CO2	WF	WF-HX	WF-CO2	Analysis of variance		
							Flour (F1)	Defatting process (F2)	F1 × F2
First scan									
∆H_gel_ (J/g_db_)	6.8 ± 0.2^ab^	7.6 ± 0.3^cd^	8.0 ± 0.3^d^	6.4 ± 0.3^a^	7.0 ± 0.1^b^	7.2 ± 0.1^bc^	*	ns	ns
*T*_p-gel_ (°C)	66.0 ± 0.1^a^	65.7 ± 0.1^a^	66.0 ± 0.1^a^	66.2 ± 0.1^a^	65.9 ± 0.4^a^	66.1 ± 0.1^a^	ns	ns	ns
∆*T*_gel_ (°C)	10.7 ± 0.6^a^	11.4 ± 0.1^ab^	11.3 ± 0.1^ab^	11.7 ± 0.5^b^	11.8 ± 0.2^b^	11.3 ± 0.6^ab^	ns	ns	ns
∆H_am-lip_ (J/g_db_)	2.8 ± 0.2^a^	3.0 ± 0.3^ab^	3.5 ± 0.3^b^	2.7 ± 0.3^a^	3.2 ± 0.1^ab^	3.2 ± 0.3^ab^	ns	ns	ns
*T*_p-am-lip_ (°C)	95.6 ± 0.8^ab^	97.0 ± 0.1^c^	96.0 ± 0.1^bc^	96.3 ± 0.4^bc^	96.5 ± 0.1^bc^	94.7 ± 0.8^a^	**	*	ns
Second scan									
∆H_ret_ (J/g_db_)	0.8 ± 0.1^d^	0.9 ± 0.1^d^	0.8 ± 0.1^cd^	0.2 ± 0.1^a^	0.5 ± 0.1^bc^	0.4 ± 0.1^ab^	**	ns	ns
*T*_p-ret_ (°C)	41.1 ± 2.4^a^	47.4 ± 1.2^b^	48.1 ± 1.4^b^	52.0 ± 2.9^b^	48.4 ± 2.8^b^	37.2 ± 2.5^a^	*	*	*
∆H_am-lip_ (J/g_db_)	4.0 ± 0.2^c^	3.7 ± 0.2^b^	4.2 ± 0.1^c^	3.3 ± 0.1^a^	3.3 ± 0.2^a^	3.6 ± 0.1^ab^	**	**	ns
*T*_p-am-lip_ (°C)	98.7 ± 0.1^ab^	99.9 ± 0.2^bc^	98.8 ± 0.2^ab^	99.2 ± 0.7^bc^	100.1 ± 0.8^c^	97.7 ± 0.3^a^	ns	**	ns

∆H_gel_ = enthalpy associated with starch gelatinization; this enthalpy is expressed in J/g db (dry basis, or total solids); *T*_p-gel_ = peak temperature for gelatinization peak; ∆*T*_gel_ = gelatinization temperature range calculated as the difference between *T*_end-set_ and *T*_on-set_; ∆H_am-lip_ = enthalpy associated with dissociation of amylose-lipid complex; ∆H_ret_ = enthalpy associated with the melting of recrystallized amylopectin; Values with different letters in the same line are significantly different at *p* < 0.05. Analysis of variance: *p < 0.05, **p < 0.01, ***p < 0.001, ns, not significant.

In the second scan, two peaks were also observed in all flours. The first peak, linked to the melting of amylopectin retrograded during storage, occurred at 46 ± 5 °C, while the second peak, associated with the reversible amylose-lipid dissociation, appeared at 99 ± 1 °C. The higher temperature of the amylose-lipid dissociation peak observed in the second scan compared to the first indicates that, following gelatinization, this complex formed a more stable structure (45). The lower melting temperature of the retrograded amylopectin compared to that observed during gelatinization is due to the amylopectin crystals formed during storage are smaller and less perfect than those in present in native granules (46). The degree of retrogradation, that reflects starch re-association during storage, varied between 3% (for WF) and 12% (for RF). These low values indicate slow staling kinetics and are like those recorded for other cereals, such as wheat (10%) (47), and some pseudocereals, such as quinoa (9–11%) and amaranth (5%) (48). However, they were significantly smaller than the values reported for other gluten-free cereal flours such as rice (60%) (49), sorghum (46%) (50) or tef (25%-34) (47). Among the samples studied, WF showed the lowest retrogradation enthalpy, four times smaller than RF. However, no significant differences were observed because of the defatting process. These findings indicate that the fiber in whole flour may hinder the reorganization of amylopectin molecules during storage, making it more difficult to form an ordered structure. The ∆H_am-lip_ increased after the second scan compared to the first by 43, 23 and 20% for the RF, RF-HX and RF-SCO2 samples, respectively. However, WF and the two defatted flours derived from it hardly varied in the second scan with respect to the first. The increase in ∆H_am-lip_ values after the second scan has been previously reported in other flours, attributed to the leakage of amylose from starch granules when temperatures exceed the gelatinization range during the first scan (44, 45). The presence of fiber in WF could hinder the formation of new or more ordered amylose-lipid complex structures after gelatinization.

## 4 Conclusion

This study demonstrated that oil extracted from canary seed flour using supercritical carbon dioxide extraction technology is a rich source of polyunsaturated fatty acids and retains significant amounts of tocopherols, unlike conventional extraction with hexane, making it a nutritionally valuable product. The defatted flours exhibited improved techno-functional properties. The water absorption capacity was higher in hexane-defatted flours due to their smaller particle size. However, flours defatted using supercritical carbon dioxide showed the highest oil absorption capacity compared to both non-defatted and hexane-defatted flours. The most significant improvement after defatting was observed in surfactant properties, including emulsifying activity and foam capacity and stability, associated with the absence of lipids and an increase in protein content. These enhanced properties make canary seeds defatted flours more valuable in food processing, as the improved absorption capacity, particularly after supercritical carbon dioxide defatting, helps to maintain texture properties while preserving nutrients. Regarding the pasting properties, defatted flours showed increased peak and breakdown viscosities, as well as a higher degree of amylose retrogradation. The rheological analysis revealed that the gels made from canary seed flours had predominantly elastic behavior, with refined flours showing a notable increase in elastic modulus after defatting with supercritical carbon dioxide. Overall, these results suggest that defatted flours, particularly those treated with supercritical carbon dioxide, have significant potential as thickening agents in food formulations due to their improved techno-functional and gelling properties, particularly relevant for thickening and structuring applications in food systems. These results underscore the opportunity to confer added value to defatted flours, by-products of the expanding industry dedicated to producing seed oils with high nutritional quality.

## Data Availability

The raw data supporting the conclusions of this article will be made available by the authors, without undue reservation.
